# Little Homes as Centres of Influence

**Published:** 1912-04-13

**Authors:** 


### BYWAYS OF MEDICAL RELIEF.*

(Contributions are invited to this column.)

### XI.—Little Homes as Centres of Influence.

The smaller the home the better the opportunity for individual treatment. Few people realise that individual treatment is what the majority of sufferers need, and seek in vain. It is the individual whom social science is seeking to benefit, and in all the strenuous schemes for national improvement to which our age has given birth the individual is the aim in view. But in the crowded tenement, in the big institution, in the massed recipients of charity or municipal aid, the individual proves elusve. He is entered in lists, warned, assisted, badgered, blamed, and encouraged. Yet his soul is a secret. It is in the little homes that forces can be brought to bear which will give that opportunity to latent qualities for which many thousands wait in vain through their whole lives.

### Curative Influences.

A magnificent impulse to curative effort in convalescent work has been started by fresh-air sanatoriums. Discarding the model of lower-grade boarding-house set by old-fashioned convalescent homes, the sanatorium aims at regenerating the patient and setting his feet on a path which must be followed in his own home after his term of residence is over. If this system were applied to the inmates of every convalescent home the era of a healthy population would draw sensibly nearer. Under the existing system, or lack of system, in homes small and large anæmic girls in process of recovery from gastric disorders may be watched sucking sweets up to the moment before bolting heavy meals, and lounging away their days in tight stays, without the smallest attempt being made to instruct them in the art of diet or exercise.

In the little homes at any rate it is possible to regard each patient as a problem to be solved. Let the owner or manager of the home realise that the period during which the patient remains in residence is a big chance in the individual life which may never happen again. It may be a sickly child; and it is no doubt true that food and fresh air may be trusted to work an improvement. But to effect a radical cure an effort must be made to discover what has produced such an unnatural being as a sickly child, and to remove the impediment which is crushing vitality. It may be a chronic invalid, in which case it is important to remember how large a factor is hope in the lives of the sick. Among those who visit the home there will be some with defective teeth; others with defective vision using imperfect or ill-adjusted glasses; others, such as babies or nursing mothers, with unhygienic garments; others with bad habits, a large majority who are ignorant of the simple rules which promote health. In all these cases a little energy, a little money, a little tactful talk will set a new current of energy stirring in ignorant, neglected, and therefore very wretched, lives. Better to limit to very few the scope of the aid afforded, and leave enough funds available to make a success of each inmate of the home.

### Educational Influences.

It is puzzling to note among our numerous uncoordinated charities how very rarely they play into each others' hands. We have many associations for teaching handicrafts to the maimed, teaching the blind to read and the dumb to speak, and yet in the homes, where there is such ample time and opportunity for giving instruction, little or nothing is done in the way of education. It would be stimulating for managers to reflect on the numerous things which could be learned in the course of a month by devoting only a couple of hours a day to the task. The mother might learn to cut out and make clothes of simple pattern for her overloaded babies. The infirm might learn lace-making or fine crochet, or flower-making, or straw-plaiting, or a hundred other things to lighten their dull hours and bring perhaps a little money into the pocket in days to come. Those whose education had been neglected would profit from reading or writing lessons. All would be cheered by the sense of getting some reward from the interval in their daily toil, while the effort to systematise the day would bring added pleasure in recreation. No doubt it costs money to pay teachers and buy materials. No doubt some would prove indifferent to chances of self-improvement; and additional trouble would have to be taken over those who responded. But the value of it is beyond computation. To turn to good account some of the long hours of convalescence or chronic disability, during which temptation, if only to gloom or giddiness, is rife, would not this be worth the effort?

### Consolatory Influences.

Passing in and out among those who are recovering from sickness, the lamentable burden of human sorrow strikes home on the observer's eye. Every one of these people has a secret source of anxiety. Hidden under their civil, patient bearing are thoughts which gnaw unceasingly. And among the factors which tend to depress and devitalise them is the want of any friend who will advise, or at least listen with sympathy. Such a friend might the head of the little home become even to those who pass but a week or two under her care. It seems there is no special art required for those who would console. A little patient attention given apparently without design to each separately, some kindly interest divested of mere curiosity—this is all sometimes that is wanted to bring the comforting assurance of friendship. The poor are sadly ignorant of the many agencies formed for their assistance, and it may be only a question of putting them into touch with some other organisation. Very often it is a story of some past blunder or wrong, grown bitter by constant brooding, and here a little brave philosophy of life spoken by one who has encountered disappointments and vanquished them will bring a new courage and hope.

